# Design, Implementation, and Evaluation of City as Text™ as a Place-Based Framework for Service-Learning in Pre-clinical Medical Education

**DOI:** 10.1177/23821205261475555

**Published:** 2026-08-05

**Authors:** Asia T. Bright, Samuel E. Neher, Marianne P. Florian

**Affiliations:** 1 Office of Professionalism, Department of Psychiatry and Behavioral Sciences, McGovern Medical School at the University of Texas Health Science Center at Houston, Houston, TX, USA; 2 Office of Educational Programs, Accreditation and Continuous Quality Improvement in the Office of the Dean, and Director of Educational Scholarship, McGovern Medical School at the University of Texas Health Science Center at Houston, Houston, TX, USA; 3 McGovern Center for Humanities and Ethics at McGovern Medical School at the University of Texas Health Science Center at Houston, Houston, TX, USA

**Keywords:** service-learning, city as text pedagogy, undergraduate medical education, professional identity formation, curriculum

## Abstract

**Introduction:**

Service-learning is a required component of undergraduate medical education and is associated with empathy, civic responsibility, and commitment to underserved populations. However, traditional experiential learning models underemphasize inequities shaping community health. City as Text™ (CAT), a place-based pedagogy, extends experiential learning by explicitly engaging learners in spatial, historical, and community-informed inquiry. This study evaluates the self-perceived effectiveness of a CAT-based service-learning curriculum in pre-clinical medical education for fostering students’ acquired knowledge of structural drivers of health, community perspective-taking, and professional identity formation (PIF), while strengthening their understanding of civic responsibility and community as a health resource.

**Methods:**

A required CAT-informed service-learning curriculum was implemented for all first- and second-year medical students (n = 480) at McGovern Medical School. Students participated in faculty-led neighborhood explorations, engaged with community organizations, and completed structured reflective narratives. Qualitative analyses of reflections were conducted *a priori* codes based directly on curriculum objectives and clustered into four domains: acquired knowledge of structural drivers of health, community perspective-taking, (PIF), and higher-order critical thinking. Comparisons were made between MS1 and MS2 cohorts.

**Results:**

Student reflections demonstrated enhanced analysis of structural drivers of health, recognition of community organizations as essential health resources, and engagement in higher-order critical thinking. MS1 students emphasized civic responsibility and non-medical drivers of health, while MS2 students exhibited more integrative, systems-oriented reasoning and explicit professional identity reflection. Across cohorts, reflections shifted from deficit-based to asset-based perspectives, positioning communities as co-educators rather than observation sites.

**Conclusions:**

Our data demonstrate that this service-learning curriculum promoted the strengths of CAT pedagogy and the values of civic medicine for pre-clinical medical students. CAT is a scalable pedagogy for service-learning in undergraduate medical education. Embedding learning within place, history, and community partnership promotes structural analysis, PIF , and equity-oriented thinking, preparing future physicians for socially accountable practice.

## Service- Learning in Medical Education

Despite changes in medical education, endorsing the ancient values of the Hippocratic Oath has remained a constant for seventy years.^1^ This oath underscores a physician’s commitment to society, prioritizing the health and well-being of communities.^1^ One meaningful way to instill and emphasize these values during training is through service-learning.

According to Stewart and colleagues, service-learning is a structured educational experience that integrates community service with preparation and reflection.^2^ It involves active participation in thoughtfully organized experiences that (1) address community needs, (2) coordinate efforts between schools and communities, (3) integrate with the academic curriculum, (4) dedicate time for reflection, (5) apply skills and knowledge, (6) extend learning opportunities, and (7) develop a genuine sense of care for others.^3^

Numerous studies have demonstrated that engagement in service-learning is associated with increased levels of empathy, a more comprehensive understanding of community health dynamics, and an enhanced commitment to the physician’s role in serving medically underserved populations.^4-6^ These qualities (empathy, community, and commitment) are central to the professional identity of a physician. Moreover, when comparing students who participate in community service work with those who do not, research consistently shows that students who engage in service hold more favorable attitudes and greater intention to work with the underserved.^7,8^ Recognizing the transformative potential of service-learning, the Liaison Committee on Medical Education (LCME) has incorporated it into accreditation standards. Standard 6.6 requires that medical schools “provide sufficient opportunities for, encourage, and support medical student participation in service-learning and/or community service activities.”^9^

### Limitations of Traditional Medical Education Pedagogy

Although service-learning is included in medical school accreditation standards, the dominant teaching methods have remained largely unchanged.^10^ According to the Association of American Medical Colleges (AAMC) Curriculum Inventory, in the 2018-2019 academic year, most medical schools relied primarily on didactic lectures.^10^ This approach risks reducing students to passive recipients of knowledge.^10^ National self-reported data on medical student behaviors have revealed that less than half of medical students report attending lecture “most of the time” or “often.”^11^ This traditional approach can lead to disengagement and a lack of intrinsic motivation to learn.^12^

In contrast, a core principle of service-learning is rooted in the educational philosophy of John Dewey, who argued that learning is most effective through experience.^13^ Dewey advocated for student-centered education, grounded in real-life contexts, where schools serve as “laboratories” for experiential learning. Consistent with the goals of civic medicine, Dewey’s pedagogy encourages intentional engagement with communities to develop socially responsible citizens. While Dewey’s philosophy of experiential learning remains foundational to service-learning, applying it in more complex contexts such as urban environments requires expansion and elaboration of the theory. The conceptualization of community interaction is abstract and does not fully address the specificity of place, space, or local context. Furthermore, Dewey’s model largely overlooks how historical legacies, power dynamics, and systemic inequities are embedded within the physical and social structures of communities, particularly within urban settings. These dynamics are key to understanding community health holistically. Moreover, these theoretical gaps often manifest in practice, with many service-learning initiatives in medical education remaining short-term, optional, or misaligned with broader educational goals.^6^ These shortcomings hinder a central pillar of a physician’s professional identity; a commitment to continuous learning and sustained community engagement.^14^

### Adoption of City as Text Pedagogy in a Service-Learning Curriculum

To address these gaps, one promising approach is the City as Text™ (CAT) (also referred to as place -based learning) pedagogy.^15^ Developed by the National Collegiate Honors Council, this method builds on and transcends Deweyan experiential learning with its general emphasis on “learning by doing.” Whereas Dewey’s model foregrounds experience as a catalyst for reflection, it offers limited guidance on how learners should interrogate the sociopolitical contexts that shape those experiences. In contrast, CAT incorporates a more rigorous framework grounded in critical spatial awareness, place-based inquiry, and structured engagement with community narratives.^15^

In our curriculum, students engage in urban environments through systematic walking explorations, mapping exercises, environmental scans, and facilitated community interactions.^15^ These activities move beyond surface-level observation by requiring learners to identify patterns in the built environment, recognize community assets, and connect these observations to health-related challenges. CAT emphasizes embodied perceptions of place, encouraging learners to physically experience the textures, rhythms, and spatial organization of neighborhoods to document what is present, missing, or contested within these spaces.^15^ Through dialogue with residents and community leaders, students gain firsthand insight into how local experiences shape health needs, resource distribution, and community-defined priorities.^15^

Building on this grounded field engagement, CAT further challenges students to interrogate the broader social, political, and historical narratives embedded in place which is an analytical dimension that targets specific reflective processes where typical Deweyan experiential learning leaves reflection unspecified.^15^ Rather than treating experience as inherently educational, CAT requires students to situate their encounters within the larger systems that produce inequity, such as zoning policies, economic disinvestment, and institutional relationships between communities and healthcare systems. By integrating Dewey’s foundational commitment to experience with an explicit focus on spatial analysis, community knowledge, and structural drivers of health, CAT offers a more comprehensive and socially attuned framework for service-learning that prepares medical trainees to understand and effectively respond to the complex forces shaping community health. This orientation echoes earlier traditions in medicine, such as house calls, where physicians engaged deeply with patients’ social contexts.^16^ Contemporary data underscore the relevance of this approach: estimates suggest that access to and quality of healthcare account for approximately 20% of health outcomes, while non-clinical factors including social support, community safety nets, childcare, and the physical environment account for up to 50%.^17^ CAT may help students recognize the essential role of these nonclinical health resources and reframe communities as integral partners in health rather than peripheral adjuncts to clinical care. For this reason, we find CAT particularly well-suited for medical education, where cultivating an understanding of health inequities, community assets, and the sociocultural dimension of care which is essential for shaping civically responsible future physicians.

### Current Study

The current study examines a service-learning curriculum in undergraduate medical education that is designed based on the CAT pedagogy. We hypothesized that implementing a CAT-based pedagogy within a service-learning curriculum would actively enhance students’ (1) ability to analyze urban structural drivers of health and identify community organizations functioning as health resources; (2) capacity to articulate community organizations’ goals and perspectives on health; (3) development of social responsibility as a core component of professional identity formation (PIF) as future physicians; and (4) engagement in higher-order critical thinking.

## Methods

### Participants

For the MS1 class, there were 240 students (109 (45%) males, 131 (55%) females) between the ages of 19-41 years. Twelve percent of students self-identified as non-traditional applicants, with one student who served in the military. For the MS2 class, there were 240 students (99 (41%) males, 141 (59%) females) between the ages of 19-39 years. Seventeen percent of students self-identified as non-traditional with two students who served in the military.

#### Curriculum

The CAT-based service-learning curriculum’s learning outcomes cover four domains: acquired knowledge of structural drivers of health, community perspective-taking, PIF, and higher-order critical thinking. The outcome-based learning objectives were to (1) assess various neighborhoods in Houston and identify the structural drivers of health (2) integrate civic responsibility into PIF , and (3) examine various cultures of health from the perspective of community organizations. First, students engaged in a didactic session with a public health professor, who explained the definition and application of service - learning and how to mitigate cognitive bias in urban neighborhoods. Then, they were transported by a medical school faculty member in small groups of 10 to a neighborhood within 20 minutes of the medical school, which is located in the largest medical center in the world. At their assigned locations, students then followed walking directions while faculty members facilitated discussions of building structures, art, language, urban green infrastructure, food sources, transportation, schools, and allied health facilities. After the walk, students visited an assigned community organization where they listened to the community organization on how they contribute and assist in the wellness of their community members. The medical students visited organizations that work on food sovereignty and nutrition, spirituality, multicultural education and understanding, housing assistance, temporary foster care, social work in support of families, alleviating childhood trauma, and support for people with dementia and their caregivers. After students completed their conversation with their community organization, they were transported back to the medical school where they debriefed with their faculty and connected community resources with the classroom objectives. Each small group will return to the same organization to offer hands-on service at least once a semester for both of their pre-clinical year totaling a minimum of four visits.

## Assessment

### Data Collection

To evaluate whether the design of the service-learning curriculum met its intended learning objectives, each student group completed a written reflection. The curriculum development team for this program created the evaluation writing prompt based on a guided critical reflection question adapted from community-engaged and service-learning pedagogical approaches in order to maximize collaboration within the student groups submitting the data.^15^ Reflections were typed and submitted electronically via Canvas. Each response was then linked to a map of Houston that displayed the geographic location of the corresponding community organization to contextualize students’ observations spatially. Approximately 48 responses were collected across all groups containing all 480 first- and second-year students. This study was approved by the Institutional Review Board of the University of Texas Health Science Center at Houston (HSC-MS-24-1081), and all procedures were conducted in accordance with ethical standards.

### Development of Coding Framework

The research team developed a coding framework through an iterative process, based upon two sets of themes: (1) the stated objectives of the service-learning curriculum and (2) the pedagogical advantages associated with CAT pedagogy. Using a directed content analysis approach, these two sets of features were used as *a priori* deductive categories to apply when coding the reflections from each student group.^18,19^ An initial set of fourteen analytic nodes (Table 1) were identified to capture each of the key learnings and insights that the curriculum was designed to promote.

**Table 1.** Nodes Grouped Into Higher-Order Clusters Aligned With the Curriculum’s Overarching Learning Objectives

Objective	Theme	Abbreviation	Definition	Codes (n)		Codes (%)	
				MS1	MS2	MS1	MS2
(1) Increase understanding of how structural drivers of health affect local communities				29	35		
1	Structural Drivers of Health	SDH	Non-medical factors that influence an individual’s health and well-being	13	10	45	29
1	Neighborhood or Local Context	NLC	Verifiable, specific attribute, and/or history for that area	10	12	34	34
1	Asset Based Mindset	ABM	Looking at the strengths of the community	4	8	14	23
1	Politics, Power, and Policy Structures	PPP	Power dynamics that effect well-being	2	5	7	14
(2) Increase understanding of community organization’s perspectives on health and availability as a health resource				44	39		
2	Well-being Factors	WBF	Six factors of well-being: Physical, spiritual, intellectual, social, emotional occupational	6	6	14	15
2	Perspectives of Community Orgs.	PCO	When the student was given information about health from the community org. Perspective	17	5	39	13
2	Organization, mission, project	OMP	States information about the mission or projects of the organization	21	28	48	72
(3) Importance of social responsibility in professional identity formation of a physician.				16	14		
3	Civic Responsibility	CRE	The students’ civic responsibility or their commitment to their community	4	1	25	7
3	Professional Identity Formation	PIF	The development process where individuals integrate the knowledge, skills, values, and behaviors of their profession	1	5	6	36
3	Socio-Cultural Awareness	SCA	The students’ understanding and appreciation of how social and cultural factors influence individuals’ behaviors, beliefs, and values	6	8	38	57
3	Inter-disciplinary Exploration	IDE	The student weaving knowledge outside of medicine	5	0	31	0
3	Reciprocity	REC	The community and student both gain something	0	0	0	0
(4) Engagement in Higher Order Critical Thinking				26	19		
4	Direct Interaction with Community	IWC	When the student was given information about health directly from the community member	0	1	0	5
4	Students Appraisal of the Environment	SAE	Students’ appraisal of the environment	26	18	100	95

### Data Coding and Consensus Process

Using this coding framework, the team proceeded with a consensus-based analysis to optimize interpretive rigor. Respective team members had varying familiarity with the CAT-based service-learning curriculum, with the student groups who participated, and with the communities that the students visited. Therefore, discrepancies between researchers’ initial coding were treated, not as interpretive errors to calculate and rectify, but rather as opportunities to clarify the implications of student reflection content in light of the curriculum’s explicit objectives and outcomes. Our consensus approach enacts Barbour’s (2001) perspective that differing interpretations among multiple coders is valuable for generating discussion, insight, and refinements to the interpretive framework.^20^

All reflection data that student groups submitted were initially coded by individual researchers. Coding was conducted manually within a word-processing document. Two members of the research team (S.N and M.F.) each independently coded half of the total dataset, after which they reviewed and assessed their counterpart’s coding. Discrepancies and interpretive questions were resolved through discussion among all three team members until agreement was reached on the final coding of each passage. This collaborative process ensured that the application of the coding framework was consistent and transparent across the full dataset and that the analysis balanced team members’ diverse areas of expertise. No passages required codes outside the *a priori* framework, suggesting that the nodes adequately captured the conceptual content of the reflections. After initial coding, the fourteen nodes were organized into four higher-order clusters that aligned with the curriculum’s overarching learning objectives (Table 1). Frequencies were calculated for each node and cluster to determine the prevalence of specific concepts within the student’s reflections (Table 1).

### Data Organization and Comparative Analysis

To enable systematic comparison across cohorts, four separate datasets were created, one for each node cluster, with each dataset further distinguished by training level (first-year and second-year students). This organization facilitated analysis and comparison of conceptually related passages, both within and across cohorts. One member of the research team (M.F.) synthesized and summarized the content within each cluster, maintaining the distinction between first- and second-year students’ reflections to facilitate comparison across cohorts. This synthesis was subsequently reviewed by a second team member (A.B.) to ensure accuracy and consistency.

This education innovation study was prepared in accordance with the DoCTRINE (Defined Criteria To Report INnovations in Education) guidelines.^21^ To supplement this, we also adhered to the SRQR (Standards for Reporting Qualitative Research) guidelines checklist when preparing the report.^22^

## Results

### Cluster 1: Structural Drivers of Health

The first cluster of thematic codes that we analyzed pertains to the community organizations’ location and surroundings and to structural factors impacting health.

#### MS1

Medical students were attuned to multifaceted historical, geographic, infrastructure, and economic features of the communities they visited. Three groups remarked on their site’s proximity to Texas Medical Center and its healthcare facilities. Two groups noted the historical significance and/or relative location of specific neighborhoods where community organizations were located within larger municipal areas. Other groups noticed development trends in the built environment and projected levels of affluence and poverty among community members.

*Emancipation Park is the oldest park in Texas. The park is dedicated to the slaves who were freed when slavery was abolished in Texas. There were over 250 thousand slaves freed during that time. After a multimillion-dollar renovation in 2017, the park gained a new recreation center. Although the community is beautiful and has all free amenities, they often go unused as social determinants place make it difficult for the community to find time to use them* (sic).

The reflections also demonstrate that medical students’ understanding of structural drivers of health was both activated and deepened during this service-learning experience. Many groups identified multiple structural drivers of health, including home and family and neighborhood resources (each mentioned twice), as well as safety, affordable housing, leisure time, access to healthy food, transportation, and access to technology (each mentioned once). Others acknowledged structural factors that hinder health and wellness, most notably having low socioeconomic status and living within ‘food deserts’ (each mentioned twice).

*The community in which Hope Farms/Recipe for Success is located is the Sunnyside community. The Sunnyside Community is a food desert and an under-resourced area, with a lower socioeconomic population. Although the organization is physically located at Sunnyside, it was not immediately clear to our group whether it is physically accessible to the Sunnyside community.*

*One hope we have for this organization is that they can make their mission a reality for the community they are physically in. The organization is not easily accessible as it is on a main road with no obvious parking locations in a non-walkable area. Further, we felt as though the organization could be more strategic in reaching and understanding its community.*

Others mentioned community members being unable to prioritize their health, overcome barriers to health from within a community, and cope with homelessness exacerbated by expulsion from public spaces of shelter (each mentioned once). One group noted that structural drivers of health need to be addressed on a case-by-case basis in consultation with trusted community partners. Overall, students recognized that circumstances beyond the control of individuals can nevertheless affect their health, an awareness that the majority of the medical school curriculum is not designed to cultivate. These situations include factors that promote wellness, as well as barriers that hinder it. Moreover, effectively addressing structural drivers of health may require community-engaged approaches.

#### MS2

Among second-year student groups, there were four mentions of geography, noting a site’s proximity to other neighborhoods and position within the greater Houston area. MS2 students noted the historical significance of specific landmarks and architecture six times. Other observations pertained to low-density urban development and its effects on pedestrians (mentioned once), the prevalence of industrial use of land, and a community’s large population of elderly people.

*[T]he Third Ward Multiservice Center is a center you can volunteer at or even rent out for events like a health fair. It’s centrally located within Houston. The DAWN program (Diabetes Awareness and Wellness Network) is free and helps participants with their diabetes! They have locations in the Third Ward, Denver Harbor, Alief and Acres Homes…*

*The Emancipation Community Center and Park was founded in 1872 to celebrate Juneteenth, and is the oldest public park in Texas. For more than 20 years, Emancipation Park was the only public park available to African Americans…*

The MS2 students’ reflections also demonstrate growing sensitivity to how structural drivers affect community health. This may be due to other elements of the MS1 medical curriculum, that introduce the framework. Building upon observations of facts and features in a neighborhood, an area’s lack of outdoor infrastructure such as parks and sidewalks was mentioned twice as a detriment to walking and exercise, and lack of access to nutritious food was mentioned five times. Additionally, lack of access to transportation was identified three times as a structural driver of health. Narrowing down to health impacts from housing, MS2 students differentiated between housing that promotes health and housing whose stability, safety, and cost impedes it (mentioned four times).

*Not many outdoor infrastructures to be able to walk outside, like sidewalks or parks. There were not a lot of places to get fresh food. It was a very industrial area. There were a lot of short-term houses rather than long-term housing. One of the things that we noticed was that places were distant from each other, and it seemed like cars were needed to travel to places in the neighborhood. Transportation was significant because it makes it difficult to obtain fresh produce and to access health care if there are not very many options for public transportation.*

### Cluster 2: Non-Clinical Health Resources

The second cluster of thematic codes concerns medical students’ understanding of how community organizations serve as non-clinical health resources.

#### MS1

At most sites, students connected with staff, including clergy (mentioned twice) and care staff (mentioned once).

*We had the opportunity to sit down and talk with Reverend Kristy about their involvement and outreach with what initially seemed to be a nonexistent unhoused community. Unbeknownst to us, there was a steady influx of unhoused people who would travel from the surrounding areas via the local bus depot for their available resources, including food and personal hygiene.*

Interestingly, some reflections described unique approaches to meeting the needs of dementia sufferers and instances when unhoused people traveled by bus to access programs and services. Overall, medical students recognized that community organizations promote health more flexibly than is often possible in a clinical setting. Community organizations use interdisciplinary and intercultural methods to alleviate child abuse and neglect, trauma due to child sexual abuse, domestic stressors while families are in crisis, family caregiver burnout, housing insecurity, and homelessness. Many clinical approaches emphasize the formal aspects of an intervention and underemphasize preserving a generous attitude toward a program’s beneficiaries. Prioritizing caring attitudes can allow for more flexible, tailored solutions to benefit a specific community. Students also recognized that a key aspect of community organization effectiveness rests on providing love, support, and nurture in the form of safe places to live; nutrition education and access; multicultural education; holistic victim advocacy; and memory care.

*This organization provides resources and opportunities for individuals diagnosed with dementia and their family members who are their main care providers. For the individuals diagnosed with dementia, they have volunteers who can come into their home and spend time with them, or they have a day center where those with dementia can come spend a day playing games or other activities while their family members can relax knowing they are somewhere safe. While this organization does not directly provide any medical care to these individuals, they are helping with the mental, social, and emotional health of all members of the family.*

However, students noted that several community organizations face obstacles. Two reflections mentioned the need for volunteers, while others noted that while an organization’s programs were well-supported with funds and staff, community members were unable to take time to engage in and benefit from programs. In another instance, program efficacy was halted when unhoused people were forcibly removed from a neighborhood where programs had been created for them. More generally, students noted that efficacy often depends on organizations being able to connect with their surrounding community in order to learn from them and gain trust. Students’ reflections showed their increasing understanding of non-clinical community health resources and the variety of programming these organizations offer to address health needs. Students recognized how these organizations work to alleviate stressors and provide support services, while also facing challenges such as volunteer shortages and barriers to community engagement.

#### MS2

MS2 students recognized that community organizations address health by alleviating, for example, lack of access to nutritious food (mentioned three times), adverse childhood experiences (ACE’s) due to family crisis or abuse (mentioned four times), and the financial and emotional burdens of cancer treatment. They also noted how organizations support specific populations that enrich community belonging, including artists and art students (mentioned three times), women and single mothers (mentioned three times), and emerging community leaders (mentioned twice). Interestingly, students recognized that connecting physicians with community-based health resources (mentioned three times) and supporting economic empowerment (mentioned twice) function as indirect but important interventions in health.

*Casa de Esperanza is a nonprofit organization that focuses on providing full-circle care for children from both unstable homes and assisting parents with unexpected events like medical emergencies. Casa gives voluntary care to children whose parents feel they cannot provide adequate care for or support the needs of their children. They provide the children with safe housing, food, education, healthcare access, and other developmental resources while working with the parents to provide them with resources in hopes of eventually reuniting parents with their children. As future physicians, we believe it is important to be aware of resources like Casa de Esperanza, as they provide vital support to patients as well. For example, patients who have medical conditions that prevent them from caring for their children (like single mothers in the ICU) can contact Casa to help give their children a safe, secure, and loving home away from home*

The community organizations that students visited offered diverse programming. One of the sites inspired a detailed description of programs and facilities for sports, fitness and recreation (mentioned ten times), while other groups site visits were focused on family crisis support services (mentioned twelve times), which included support for children and their families, as well as for sick people and their caregivers. Students also described community organizations providing education programs and resources, nutrition access, and community gathering spaces (each mentioned seven times); career development programs (mentioned six times); health education (mentioned five times); and affordable and appropriate housing (mentioned five times). Additionally, some of these same organizations also provide free healthcare and counseling/therapy services to their beneficiaries (mentioned four times) and social work services for veterans (mentioned once). MS2 students’ reflections demonstrate both the breadth of community health resources available and the students’ own capacity to recognize how these varied programs address various aspects of health.

*Combined Arms is an organization dedicated to supporting military veterans and their families by connecting them with over 300 regularly vetted organizations that address their specific needs, focusing on the social determinants of health. Integrated into hospital systems around Houston, Combined Arms works closely with social workers to ensure eligible patients are seamlessly filtered into their network if they choose. The organization prioritizes the most immediate needs of veterans using the Life Stat 360 survey, a simple yet effective tool that maps out the resources required to support them throughout the process.*

However, students noted that community organizations face significant obstacles. Reflections mentioned low attendance for events, imminent gentrification and economic displacement threatening organizational stability, and isolated locations with lack of transportation limiting community access. These observations suggest that students are developing a more nuanced understanding of community health work and recognizing not only the value these organizations provide but also the systemic challenges that affect their ability to serve their communities effectively.

### Cluster 3: Social Responsibility and Professional Identity Formation

The third cluster of thematic codes that we analyzed as a unit relates to medical students’ recognizing social and community responsibility as part of physicians’ professional identity. The students’ recognition of their own role in connecting patients with community health resources is a key feature distinguishing passages coded within Cluster 1 and Cluster 3.

#### MS1

Students expressed an awareness of their community responsibility in three basic ways: commitment to understanding community as the context of people’s health, recognizing specific health impacts of socioeconomical variables on potential patients, and expressing humanistic perceptions of other people as intrinsically worthwhile and valuable.

*…[W]e encourage our fellow students who will inevitably be integrated into their respective communities to ask before assuming. Because even in a city like Bellaire [a high SES community], it’s impossible to truly understand the needs, wants, and access a community may truly have.*

Another group emphasized the way that an organization educated the public about cultural heterogeneity and complexity (mentioned twice). Several groups also highlighted the importance of connecting with trustworthy local community members when trying to enhance community health (mentioned three times).

*We propose that a community liaison who is a member of the neighborhood would benefit this organization, giving valuable insight into the community and being a connection point to other community organizations.*

*It is important to address the social determinants of health on a case-by-case basis. What this looks like in practice is having a trusted community partner, you can ask about which resources would be best to connect your parents to*

Another way students articulated a developing sense of professional responsibility as future physicians was through their growing awareness of socioeconomic disparities and their implications for patient care. Students demonstrated increasing sensitivity to the socioeconomic conditions influencing the health of the communities they visited and reflected on how these factors would shape their roles as practicing physicians. In neighborhoods surrounding community organizations, students recognized the presence and health impact of wealth disparities within the local community (mentioned twice).

*Overall impressions of ChristChurch Presbyterian: Strong disparity between a wealthy neighborhood and the unhoused populations. Minister Kristy told us about the strong initiative by local police to keep the unhoused out of the community.*

Several students noted that resource scarcity, limited transportation options, and the absence of safe walking infrastructure posed significant barriers to community organizations’ efforts (mentioned twice), prompting reflection on how they themselves might eventually address these challenges in their future clinical practice. The thread that ties these realizations together consists of humanistic values, that is, the recognition that humanity itself and individual human beings have an intrinsic value and dignity. Medical students expressed several humanistic values in their reflections.

*The staff refer to those diagnosed with dementia as “clients” instead of patients, shifting the focus away from their diagnosis. But when they do speak about someone’s diagnosis, they always use person-first language and refer to them as a person with dementia.*

One group shared that they valued the unique opportunity to spend time with community members. Another expressed the belief that all people deserve to be cared for and deserve access to resources necessary for health. On four different occasions, groups expressed that respecting the dignity of the people one seeks to help is vitally important and mentioned various practices for upholding that principle.

*We hope others know from this [medical student] community that you can give back and provide aid to a community while still maintaining community dignity. With service, it is important to emphasize respect and get people in the door to further education. Everyone deserves care and access to resources.*

Collectively, these reflections reveal students’ emerging understanding that effective community engagement requires both recognizing structural challenges and upholding the fundamental dignity and worth of all community members.

#### MS2

The webs of connections MS2 students write about fall on a spectrum from adjusting medical practice to meet a community’s linguistic needs, on one hand, to redefining health and healthcare as fundamentally interrelated with community and community-based organizations, on the other. Several reflections centered on humanistic values, particularly regarding culture and connection. Two groups mentioned culture and related concepts, though with different emphases: one expressed an abstract understanding of culture and its significance to people, while another noted their host organization’s role in preserving and transmitting culture and acknowledged how development and economic displacement might “overshadow” it.

*Culture can be defined as what a group of people can identify with, and which encompasses much of their daily lives…… We really needed to know about the resources they could offer. As medical students, we are better able to understand how we can use those resources to be able to provide healthier recipes.*

Relatedly, a group visiting an organization with an arts program observed that art serves multiple functions such as transmitting local culture, fostering community, and teaching visitors about wealth disparities. Some MS2 students expressed a strong desire to connect meaningfully with the populations they would serve. One poignant reflection shared students’ eagerness to understand the experiences of an organization’s beneficiaries to better discern when and to whom they should recommend programs:

*I would like to interact with veterans who have benefitted from Combined Arms, as understanding the specific changes in their lives would enhance my ability to recommend the organization to patients confidently. The firsthand knowledge would help address patient concerns and provide them with reassurance through personal stories of success.*

Students also recognized the interdependence between clinical practice and community knowledge. Two groups noted that familiarity with a community could enhance their medical practice. One emphasized that understanding communities was essential for understanding patients, while another recognized that knowing about non-clinical health resources could help them support patients and their loved ones. This latter group further envisioned deeper partnerships between their clinical work and community organizations, such as collaborating to host community-based clinics at the organization’s facility. This suggests that students are beginning to conceptualize medicine not as an isolated clinical career, but as responsive and relational practice embedded within communities.

This developing perspective is enhanced by students’ awareness of specific contextual and socioeconomic realities. They demonstrated contextual awareness by noting practical considerations such as the presence and prevalence of Spanish-speaking participants in organizational programs and the corresponding need for translation services (mentioned by two groups). One group also recognized socioeconomic realities, explaining their host organization’s work in terms of addressing the obstacles to career achievement that women face due to inequality. These observations demonstrate that students were simultaneously envisioning practicing community-engaged medicine while attuning the particular circumstances and structural challenges that shape people’s health in specific communities.

### Cluster 4: Student Subjective Observations

The fourth cluster of thematic codes relates to medical students’ opinions and impressions of the communities and the organizations they visited for service-learning. Students expressed their various impressions in the form of reactions, realizations, and appraisals. While some of this content relates to other clusters, a key feature distinguishing its coding is the subjective, interpretive, and/or evaluative framing of statements. When a passage of text appeared to be a student’s opinion, it was coded within cluster 4, whereas when information was expressed as if it were objective or fact-based, it was not.

#### MS1

Many groups shared positive impressions of their experiences. Three reflections expressed warm appreciation for the beauty and quality of local surroundings, while three groups complimented the work and helpfulness of organization staff and volunteers. Eleven of the student reflections expressed a high opinion of the quality and/or extent of the organizations’ efficacy, programs, services, and exhibits.

*The neighborhood provides a safe and structured environment. Their facilities include clean, well-organized homes, large play areas, and a food pantry to allow the kids to just be kids. Casa exudes a culture of compassion and stability that emphasizes the love these parents have for their children and the lengths they will go to keep them safe.*

Reflecting on affective experience, one group shared that they enjoyed their site visit, while another appreciated their site’s programs as being especially appealing to medical students like themselves.

*Through a variety of public programs and exhibitions, the Texas Asia Society enriches understanding of Asia, promotes meaningful dialogue, and sparks innovative ideas across art, culture, business, and education. Asia Society Houston is particularly inviting for medical students, offering free memberships that make it a welcoming space to unwind or study. With a café and ample open areas, it’s the perfect spot for both relaxation and productivity.*

Alongside these positive impressions, students also shared constructive observations and recommendations. They noticed opportunities for improvement and sometimes recommended strategic changes in their reflections. Students’ site-focused constructive feedback reflected two primary themes. First, students critiqued the limited resources available in the surrounding community, which they perceived as negatively affecting both community health and the organization’s capacity to provide support. Second, several student groups observed that community organizations would benefit from stronger connections with the communities they serve to enhance their overall impact (mentioned three times). In relation to this concern, some students proposed strategies for enabling organizations to better assess community needs and engage trusted community partners.

Students also turned their critical lens on themselves, recognizing their own need for additional education and information about their sites. Two of the student groups noted that they lacked sufficient information about their organization’s programs and the surrounding neighborhood to be able to evaluate them. Other groups shared that they had adjusted their preconceptions after discovering that the affluent surface appearance of the built environment had led them to underestimate the community’s needs (mentioned twice).

*At first,* [the neighborhood] *appeared very affluent: beautiful parks, well-maintained… Walking along the streets, one could assume the people have accessible health care, are insured, have access to well-funded education, and have readily available public transportation… it would have been hard to guess that this was not the reality.*

These reflections suggest students were using their subjective experiences to identify community organizations’ strengths and obstacles to non-clinical health promotion and to evaluate their own strengths and shortcomings for understanding and potentially facilitating this type of intervention. While not a majority reaction, we consider such self-awareness a valuable outcome that can motivate medical students to seek deeper contextual understanding and to think critically about strengthening capacities for improving community health beyond clinical settings.

#### MS2

MS2 students’ reflections expressed both personal responses to their experience as visitors and their impressions of the quality and accessibility of services being offered to community beneficiaries.

Groups’ positive reactions emphasized the welcoming nature of these organizations and the quality of their work. Multiple groups described receiving a warm welcome from their host organizations (mentioned four times). One group explicitly mentioned their enjoyment of the experience, suggesting that these visits were not merely educational obligations but genuinely enriching encounters. Another group said that meeting engaged community members motivated students to engage more deeply themselves. Beyond this interpersonal warmth, students appreciated the practicality of facilities and programs (mentioned six times), particularly noting when programs were accessible and affordable to the communities they served (mentioned three times). Several reflections conveyed a sense of pleasant surprise and students noticed diverse, vibrant culture in the communities they visited (mentioned twice) and expressed being impressed or having their expectations exceeded (mentioned twice).

*The community is so much more than what it might look like from the outside.*

*Seeing how important a sense of community was to the individuals at the center really made me want to come back and volunteer one day. It was great witnessing the sense of community and pride the ladies and service center people had. I enjoyed listening to how proud the community was of their progress. They did a good job of making us want to engage and support.*

MS2 students also noticed when the effectiveness of community programs was impeded by structural factors, focusing less on their host organizations’ limitations and more on the broader structural challenges faced by residents and organizations in the surrounding neighborhoods. Two groups noticed signs of low income and scarce resources, including limited access to education, food, and transportation. More concretely, three groups observed low-density development, lack of sidewalks, and related factors impeding pedestrian movement and making residents reliant on cars. Other observations included industrial development (mentioned once) and recognition that nearby housing was not likely to be stable or long-term (mentioned once).

*Based on our initial observations, it appeared that the community was primarily lower income and lacked easy access to sources of food, education, and transportation. Moreover, a lack of pedestrian infrastructure makes traveling even around the neighborhood more challenging*

There was also enthusiasm for an organization’s work coupled with awareness of environmental barriers. These pairings suggest that students are developing a more sophisticated understanding of the collective work and the shared challenges of community health. They simultaneously appreciate the vital work that organizations do while recognizing the structural constraints within which these organizations operate. This tension affects the health of the communities that they serve; communities of people who may one day seek healthcare from these future physicians.

## Discussion

Consistent with our hypotheses, the CAT approach functioned as a robust pedagogical framework for service-learning in undergraduate medical education. Participation in a CAT-informed curriculum enhanced students’ (1) ability to analyze urban structural drivers of health and identify community organizations as health resources; (2) capacity to articulate community organizations’ goals and perspectives on health; (3) development of social responsibility as a component of emerging professional identity; and (4) engagement in higher-order critical thinking. Collectively, these findings demonstrate that CAT offers a powerful means of situating medical education within the lived realities of communities and advancing key competencies essential to the practice of socially responsive medicine.

These findings also extend the existing service-learning literature in medical education. Prior studies have largely reported outcomes such as increased empathy, commitment to underserved populations, and civic responsibility.4-8 In contrast, our findings suggest that CAT may additionally support learners’ ability to analyze structural determinants of health, engage with community-defined perspectives on health, and connect these experiences to emerging professional identities. By examining these processes, the present study contributes a place-based framework for understanding how service-learning may facilitate deeper structural and community-oriented learning.

While both first-year (MS1) and second-year (MS2) medical students demonstrated acquisition of these learning outcomes, notable differences emerged in how students integrated new knowledge into their existing cognitive frameworks. MS2 students consistently demonstrated more integrative and systems-oriented reasoning. This difference is not unexpected, as MS2s prior full year of medical training which introduced non-medical drivers of health and the importance of community health resources, whereas MS1s were just beginning these courses.

### Understanding Structural Drivers of Health Through Place-Based Learning

Findings from Cluster 1 (structural drivers of health, hypothesis 1) illustrate CAT’s effect in eliciting and strengthening students’ understanding of structural drivers of health within urban environments and the health resources that community organizations represent. Students demonstrated understanding of community organizations’ efforts within broader historical, socioeconomic, and infrastructural contexts. Themes within this cluster included awareness of place, community assets, and power structures. Students frequently referenced historical, geographic, and infrastructural characteristics of neighborhoods. Coding frequencies of MS1 and MS2 students’ reflections indicate consistent and growing awareness of place (See Table 1). For example, at the site of Emancipation Park, students, specifically MS1’s, identified how historical narratives of emancipation, segregation, and civic investment continue to influence contemporary community health and access to resources. These findings suggest that CAT enables even early learners to connect history and power with health. Asset-based mindsets, while less frequently articulated than deficit-focused ones overall, were more prevalent among MS2 students’ reflections (See Table 1). This suggests a developmental shift toward recognizing community strengths, rather than focusing solely on needs and challenges. Within our sample, MS2 students were more likely than MS1s to mention power dynamics and policy-level influences, however, these topics were not frequently referenced overall. We were also interested in students’ ability to integrate their insights and reasoning when considering multiple structural determinants of health. While MS1 students were attuned to the impacts of specific structural determinants of health, MS2 students more frequently articulated how multiple factors in the structural and social environment could combine and interact to shape health outcomes, reflecting a more systemic perspective.

#### Learning With, Rather than About, Community Organizations

Cluster 2 (community organizations. Perspectives, hypothesis 2) highlights CAT’s strength in cultivating perspective-taking and community-engaged learning. Unlike Deweyan models that focus primarily on the learner’s individual experience, CAT emphasizes learning with and from communities, positioning community members as holders of lived expertise and localized knowledge.^15^ Both MS1 and MS2 students demonstrated an ability to understand health from the standpoint of the community organizations they visited, distinguishing how organizations define their own missions and values rather than relying on externally imposed priorities. This was the most dominant finding from this cluster, particularly among MS2s (see Table1). Students’ reflections commonly detailed organizational missions, ongoing projects, and service models, indicating that students recognized community organizations as legitimate and essential health resources. This type of insight crucially dismantles outsider bias and encourages learners to respect a community’s self-definition and embedded understanding of health. Differences again emerged by training level. MS1 students tended to focus on learning which services organizations provide, whereas MS2 students more frequently reflected on how community perspectives could or should be integrated into the clinical care system to support seamless, holistic care. This progression may be due to MS2s more advanced formation of their professional identity.

More broadly, these findings suggest that CAT may contribute something distinct from traditional service-learning approaches. Whereas many service-learning models emphasize participation and reflection, CAT provides learners with an explicit framework for analyzing how place, history, power, and community knowledge shape health experiences. By structuring learners’ attention toward these dimensions, CAT may help operationalize concepts such as structural competency and community-engaged practice that are increasingly emphasized within medical education.

#### Implications for Professional Identity Formation (PIF)

CAT explicitly situates learners within relational and structural contexts, prompting students to envision themselves as future physicians practicing community-engaged medicine. Students were not only observing community organizations but also reflecting on how structural challenges shape health and how their future clinical roles might respond to these realities.

Findings related to Hypothesis 3, incorporating social/civic responsibility into professional identity (cluster 3), indicate that while neither MS1 nor MS2 students explicitly referenced PIF, qualitative themes revealed early engagement with values central to socially accountable medical practice. Interestingly, the code Civic Responsibility was more prevalent among MS1 students (see Table1). This may suggest a shift from declarative expressions of responsibility among MS1s to more integrated or reflective professional identity narratives among MS2s. This is further supported through the finding that Professional Identity Formation codes increased substantially among MS2 students, highlighting a developmental progression in which learners increasingly articulated how community engagement influenced their identity as future physicians. MS1 students emphasized learning directly from community members, the importance of people-first language, preserving human dignity while offering assistance, valuing community voice in health decision-making, and avoiding assumptions. MS2 students highlighted the importance of firsthand knowledge from community members to inform clinical recommendations, including culturally appropriate resources such as healthier food options. These reflections suggest that CAT supports implicit PIF by exposing students to models of community-engaged practice that foreground humility, partnership, and respect.^15^

Notably, socio-cultural awareness emerged as one of the most prominent themes, particularly among MS2 students. Learners demonstrated growing appreciation for how cultural, social, and contextual factors shape patient experiences, behaviors, and health outcomes. Complimentary, interdisciplinary exploration was present primarily among MS1 students, suggesting early experimentation with integrating perspectives from outside their medical training. No explicit references to reciprocity were identified in either cohort, indicating a gap in students’ articulation of mutual benefit between learners and communities. Nonetheless, students articulated attributes they sought to integrate into their developing professional selves. These dispositions toward community-engaged practice are further supported by CAT’s capacity to develop the analytical skills students need to act on them.

#### Fostering Higher-Order Critical Thinking and Asset-Based Perspectives

Finally, findings supporting Hypothesis 4 demonstrate that CAT promotes higher-order critical thinking by encouraging students to hold multiple, sometimes competing, perspectives simultaneously. Although the curriculum was intentionally designed to promote direct learning from community members, students rarely referenced these interactions in their reflections, with only one explicit mention identified among MS2 participants. Student Appraisal of the environment dominated this objective, accounting for nearly all codes across both cohorts. Students consistently demonstrated higher-order critical thinking through evaluation, interpretation, and contextual analysis of community environments, indicating strong engagement with reflective and analytical processes. Students expressed appreciation for the essential work of community organizations while also recognizing the structural constraints such as funding limitations, policy barriers, and systemic inequities within which these organizations operate. This ability to grapple with complexity is central to deep learning and essential for future physicians who will serve structurally marginalized populations.

An additional strength of CAT lies in its capacity to shift students’ perspectives from deficit-based to asset-based understandings of communities.^15^ Although students initially identified more challenges than strengths, reflections increasingly acknowledged community capacities, networks, cultural richness, and resilience. Through walking neighborhoods, engaging with residents and or community leaders, and critically reflecting their observations, students began to recognize communities as assets rather than problems to be fixed. This aligns with principles of appreciative inquiry and suggests that CAT may offer an alternative to problem-based approaches that risk reinforcing deficit narratives.

Taken together, these findings indicate that CAT is a particularly well-suited pedagogy for service - learning in undergraduate medical education. By embedding learning within place, history, and community partnership, CAT facilitates structural analysis, perspective-taking, early PIF, and higher-order critical thinking. Importantly, CAT supports developmental learning across training levels and encourages students to conceptualize communities as co-educators and essential contributors to health. As medical education continues to prioritize social accountability and structural competency, CAT offers a scalable and theoretically grounded framework for preparing future physicians to practice community-engaged, equity-oriented care.

These findings may have implications beyond our institution. Medical schools nationally continue to seek effective approaches for meeting educational priorities related to service-learning, community engagement, structural competency, and social accountability. Although accreditation standards encourage meaningful community engagement, educators often have limited guidance regarding pedagogical frameworks capable of connecting community experiences to specific learning outcomes. CAT may offer one such framework by linking community engagement activities to structured observation, critical analysis, and reflective learning processes that support these educational goals.

Although the specific neighborhoods and community organizations described in this study are unique to Houston, the educational mechanisms underlying CAT pedagogy are not location dependent. Place-based inquiry, structured observation of the built environment, engagement with community-defined expertise, and critical reflection can be implemented across urban, suburban, and rural settings. Accordingly, the transferability of this work lies less in the specific sites visited and more in the pedagogical framework itself. As medical schools increasingly seek approaches to teach structural competency, social accountability, and community engagement, CAT offers a theoretically grounded model that can be adapted to diverse institutional and geographic contexts.

### Strengths and Limitations

A key strength of this study is that it evaluates a clearly articulated pedagogical framework rather than a single community partnership or isolated service experience. The curriculum was implemented across two entire medical school cohorts and multiple community settings, allowing examination of recurring learning patterns across diverse organizational contexts. This breadth supports the transferability of the educational observations despite the single-institution design.

As a qualitative educational study conducted at a single institution, the findings should be interpreted as contextually situated rather than statistically generalizable. However, the detailed description of the curriculum, learning objectives, community partnerships, and implementation processes allows readers to assess the transferability of the CAT framework to their own educational settings.The curriculum engaged students with varying levels of prior exposure to, and appreciation for, community engagement. This structure may provide opportunities for growth over time, particularly for students whose initial perspectives reflect limited experience with or understanding of the communities they serve. At the same time, we acknowledge that such perspectives are shaped by multiple factors, including prior life experiences and institutional admissions processes, which vary across medical schools. Another strength of this study was that student groups were assigned to both impoverished and non-impoverished urban areas of Houston. Health inequities and structural drivers of health are not exclusive to poverty. By working in more resourced neighborhoods, students were able to observe how factors such as stress, chronic disease, and lifestyle-related conditions affect health outcomes. The CAT pedagogy offered a structured framework through which students could begin to critically reflect on these observations, although the depth and maturity of these reflections varied.

One limitation of this study is that service - learning is a longitudinal approach. However, the current study represents data from one timepoint. As a result, the findings reflect early-stage reflections, which may include incomplete, evolving, or, at times, unrefined understandings of social and cultural complexity. Some student comments suggest limited self-awareness or reliance on assumptions, underscoring the developmental nature of these competencies.

Second, the study does not include detailed contextual data regarding students’ prior experiences, backgrounds, or admissions characteristics, which may influence their perspectives and engagement. Potential relationships between demographic characteristics (e.g., age or non-traditional student status) and learning outcomes within this curriculum could not be meaningfully examined, as each reflection represented the collective perspective of a small student group rather than individual student responses. We will expand data collection in future studies to evaluate the longitudinal impact of the CAT pedagogy on service-learning outcomes and narrow our unit of analysis from small student groups to individual students. Lastly, the study relied exclusively on reflective narratives without objective or comparative measures, limiting the ability to draw conclusions regarding the curriculum’s effectiveness beyond students’ perceived outcomes.

### Future Directions

Lessons learned from this pilot implementation included the importance of asking more targeted and probing reflection questions, as well as collecting responses at the individual rather than group level to better capture variation in student characteristics and experiences. The pilot phase also highlighted the value of incorporating additional community sites to broaden student exposure to diverse settings and perspectives. Future research should examine CAT implementation across multiple medical schools, geographic regions, and community contexts to determine which findings are robust across settings and which remain context dependent. Finally, the findings underscored the need for more comprehensive facilitator training to promote consistency in discussion quality, reflection depth, and student engagement across groups.

Although the current study provides initial evidence to support the value of CAT pedagogy in undergraduate medical education, future research may consider incorporating it in graduate medical education for residents and fellows as well as for faculty development. This approach highlights community partnership as a core tenet of medicine, regardless of one’s stage in career development and the importance of longitudinal service. Moreover, given that medicine is inherently a team-based career, future research should examine interprofessional teams (e.g., nursing and medicine) using CAT service - learning.

## Conclusion

To our knowledge, this is the first study to explore the application of CAT pedagogy within a service-learning framework specifically tailored for medical students. More broadly, this study suggests that place-based pedagogies may offer an effective mechanism for integrating structural determinants of health, community engagement, and PIF within pre-clinical medical education.These elements may contribute to shaping knowledge acquisition and goal orientation during undergraduate medical training. Given the descriptive nature of our findings, our results suggest that expanding curricular time dedicated to educational experiences outside the traditional classroom setting, may be beneficial, particularly when complemented by place-based pedagogy. Additionally, service-learning opportunities may benefit from intentionally incorporating the perspectives of community organizations, which could help illustrate the dynamic interplay between clinical systems and the broader social and community infrastructures in which they operate, while increasing student awareness of the diverse health resources available to patients.
